# Toxicity of the high-dose chemotherapy CTC regimen (cyclophosphamide, thiotepa, carboplatin): the Netherlands Cancer Institute experience

**DOI:** 10.1038/sj.bjc.6601001

**Published:** 2003-06-10

**Authors:** J G Schrama, M J Holtkamp, J W Baars, J H Schornagel, S Rodenhuis

**Affiliations:** 1 Department of Medical Oncology, The Netherlands Cancer Institute, Plesmanlaan 121, 1066 CX Amsterdam, The Netherlands

**Keywords:** high-dose chemotherapy, solid tumours, peripheral blood progenitor cell transplantation, toxicity

## Abstract

High-dose chemotherapy (HD-CT) has a role in the potentially curative treatment of several tumours. The relative efficacies of the different regimens have not been studied in comparative trials, but it is clear that toxicities differ significantly between them. We analysed the immediate and long-term toxicity in the first 100 consecutive patients treated with the CTC regimen (cyclophosphamide 6000 mg m^−2^, carboplatin 1600 mg m^−2^ (or 20 mg ml^−1^ min under the curve (AUC)) both as daily 1 h infusion, thiotepa 480 mg m^−2^ as twice daily 30 min infusion, all divided over 4 consecutive days) followed by peripheral blood progenitor cell reinfusion (PBPC-Tx). Most patients had high-risk (*n*=86) or metastatic (*n*=4) breast cancer, or a germ cell tumour (*n*=8). Two patients (with a medulloblastoma and an aesthesioneuroblastoma, respectively) received CTC as off-protocol salvage regimen. The main toxicity was bone marrow suppression. Most patients had PBPC-Tx with granulocyte colony-stimulating factor (G-CSF), and the median time to neutrophil count 500 × 10^6^ l^−1^ and platelet count >20 × 10^9^ l^−1^ without transfusion independence was 10 (range 8–25) and 13 (8–60) days, respectively. The toxic death rate was 1%. Other frequent toxicities were neutropenic fever requiring antibiotics (*n*=65), central catheter-related infection (*n*=12) or a bleeding episode (*n*=48), mostly epistaxis (*n*=26). Reversible cardiac toxicity was seen in six patients and pulmonary events occurred in seven patients (infection (*n*=6), embolism (*n*=1)). Grade 3–4 gastrointestinal toxicity was frequent: nausea and vomiting 55%, diarrhoea 28% and mild liver toxicity (transaminase elevations) 9%. One patient pretreated with cisplatin had a kidney transplantation 8 years after HD-CT. Late complications included reversible radiation pneumonitis (*n*=12) and chronic heart failure (*n*=2). We found five second solid malignancies and two myelodysplasias. In conclusion, the CTC regimen is associated with a moderate, mainly reversible, toxicity. Future studies need to compare the efficacy and toxicity of the different HD-CT regimens.

Many thousands of patients with solid tumours and lymphomas have received high-dose chemotherapy (HD-CT) with autologous blood progenitor cell rescue in the past decades (Antman *et al*, 1997; Rosti and Ferrante, 2001). While this treatment modality has been accepted as the standard of care in the salvage setting for certain malignant lymphomas and germ cell tumours, its role in the management of frequently occurring tumours such as breast or ovarian cancer is still unclear, as the results of randomised phase III studies are still pending. For high-risk breast cancer, for example, preliminary data have now been reported from nine studies involving over 3000 patients (Rodenhuis, 2000). The first statistically meaningful analyses of these studies and of yet unreported studies would hopefully be evaluated in the 2005 Oxford Overview.

While the relative efficacies of the different high-dose regimens have not been studied in comparative trials, it is clear that toxicity differs, and that certain regimens are associated with considerable morbidity and even with mortality. The STAMP-I regimen, for example, which incorporates cisplatin, cyclophosphamide and BCNU, is associated with a 12% mortality rate, mainly resulting from (BCNU-related) lung toxicity (Antman *et al*, 1997; Rosti and Ferrante, 2001). The widely employed STAMP-V regimen, a combination of cyclophosphamide, thiotepa and carboplatin (CTC), has been reported to be well tolerated (Antman *et al*, 1992). STAMP-V has been employed in several large randomised studies in breast cancer (Bergh *et al*, 2000; Stadtmauer *et al*, 2000), none of which showed a (progression-free) survival advantage for the patients receiving this treatment.

A regimen similar to STAMP-V, called CTC, has been reported from our institution (Rodenhuis *et al*, 1992). It contains the same agents as STAMP-V, but the carboplatin dose is twice as high and the agents are administered as short-term infusions rather than as continuous infusions. CTC has been used in several randomised and nonrandomised studies in high-risk (van der Wall *et al*, 1995; Rodenhuis *et al*, 1996, 1998, 1999, 2000; Schrama *et al*, 2002) and in stage IV breast cancer (Schrama *et al*, 2001; De Gast *et al*, 2002) and, moreover, in the salvage therapy of germ cell cancer (Rodenhuis *et al*, 1996, 1999).

Since CTC is associated with only moderate toxicity and recent results by our group indicate that this toxicity can even be reduced further when ‘Therapeutic Drug Monitoring’ is employed (Huitema *et al*, 2001), we believe that future studies will need to compare the efficacy and toxicities of CTC to that of other high-dose regimens. To facilitate the design of this type of comparison, we have analysed the immediate and long-term toxicities of CTC in the first 100 consecutive patients who received this regimen in our institution. For this patient group, a median follow-up of almost 5 years is available with a lead follow-up of over 11 years (137 months).

## PATIENTS AND METHODS

### Patients

We analysed the first 100 consecutive patients treated with the high-dose CTC chemotherapy regimen, who were treated between 1989 and 2000 in our institution. The CTC regimen is a widely used regimen and besides patients with breast cancer we analysed even patients with germ cell tumours and other tumours to study whether the pretreatment was important for toxicity.

Of these patients, 86 had high-risk breast cancer and were included in one of two randomised adjuvant chemotherapy studies (Rodenhuis *et al*, 1998, 2000). Four patients with metastatic breast tumours were treated in a pilot study. Six of eight patients with germ cell tumours were treated in another previously published study (Rodenhuis *et al*, 1999). The two patients with either aesthesioneuroblastoma or a medullablastoma received the regimen as part of an off-protocol salvage regimen (Table 1

**Table 1:** Patient characteristics

**Characteristics**	**All patients**
No. of patients	100
Median age (years) (range)	46 (21–57)

*Type of tumour*	
Germ cell	8
Breast cancer	
Stage II–III	86
Stage IV	4
Asthesioneuroblastoma	1
Medulloblastoma	1

*Prior to chemotherapy*^a^	
Cisplatin-based	8
Anthracyclin-based	1

*Prior to surgery*	
Mastectomy	73
Breast-conserving surgery	15
Orchidectomy	9
Other	5^b^

*Prior to radiotherapy*	
Breast/parasternal	2
Brain	1
Other	1

a Chemotherapy before enrolment in the treatment protocol.

b Two patients had a sternotomy, two patients had a laparotomy and one patient had a craniotomy, all because of metastatic disease.

). Written informed consent was obtained from all patients at enrolment in the studies, and the Committee on Medical Ethics of The Netherlands Cancer Institute approved all these studies.

### Treatment regimen

#### Induction therapy regimen and collection of progenitor cells

The treatment regimens are summarised in Table 2

**Table 2:** Treatment regimens

	**No. of patients**	**Treatment regimen**
Germ cell tumour	8	Carboplatin/etoposide^a^

*Breast cancer*	90	
Stage II–III		
Adjuvant	55	5 × FE_90_C^b^→CTC→RT and tamoxifen
Neoadjuvant	31	3 × FE_120_C^b^→surgery→1 × FE_120_C→CTC→RT and tamoxifen
		
Stage IV	4	Methotrexate and/or anthracyclin-containing regimen→CTC
Asthesioblastoma	1	Cisplatin/ifosfamide/etoposide→CTC
Neuroblastoma	1	2 × EVAIA/2 × BEP/1 × ifosfamide/etoposide→CTC

a Two courses carboplatin 800 mg m^−2^ day 1 and etoposide 500 mg m^−2^ days 1–3.

b FE_90_C=5-fluorouracil 500 mg m^−2^, epirubicin 90 or 120 mg m^−2^ and cyclophosphamide 500 mg m^−2^ all on day 1.

. The procedures of bone marrow harvesting and leucoytaphoris have been described previously (van der Wall *et al*, 1995; Rodenhuis *et al*, 1996, 1998, 1999, 2000).

#### High-dose regimen

All patients received the same high-dose regimen consisting of one cycle of cyclophosphamide 6 g m^−2^ as a daily 1 h infusion, thiotepa 480 mg m^−2^ as a twice daily 30 min infusion, and carboplatin 1600 mg m^−2^ (in case of abnormal kidney function, the carboplatin dosage was based on an area under the curve (AUC) of 20 mg ml^−1^ min) as a daily 1 h infusion, all divided over 4 consecutive days. Mesna (500 mg) was administered six times daily for a total of 36 doses, starting 1 h prior to the first cyclophosphamide infusion. The peripheral blood progenitor cell reinfusion (PBPC-Tx) was performed 2 days after the end of the chemotherapy. The prophylactic use of antiemetics and antibiotics of the separate studies have been described elsewhere (Rodenhuis *et al*, 1992, 1996, 1998, 1999, 2000; van der Wall *et al*, 1995). Briefly, all patients received antiemetics prophylactically, consisting of dexamethasone and serotonin antagonist and/or metoclopramide. Antibiotic prophylaxis consisted of oral ciprofloxacin 1000 mg (divided over two daily gifts) and oral amphotericin B (2000 mg divided over four daily gifts). Cultures of throat, nose, stool or perineum and central venous catheter (CVC) were performed twice a week. In the first CTC courses, cultures of the CVC were performed every day. As prophylaxis for infection with Gram-positive bacteria, patients either received penicillin G 1 × 10^6^ IU q.i.d. or roxithromycin 150 mg b.i.d. orally. The large majority of patients received acyclovir either orally or intravenously to prevent herpes reactivation. In case of fever, antibiotic treatment was started empirically, either with vancomycin 500 mg q.i.d. and ceftazidime 2 g three times daily or with teicoplanin 400 mg and ceftriaxone 2 g once daily. All but 15 patients received daily granulocyte colony-stimulating factor (G-CSF) injections from the day of transplantation until the WBC count exceeded 5 × 10^9^ l^−1^. Irradiated leucocyte-free red blood cells and platelets were given to maintain haemoglobin and platelet count above 5.5 mmol l^−1^ and 10 × 10^9^ l^−1^, respectively.

### Toxicity

The toxicity data were extracted from patient records and from databases associated with various studies in which the patients had participated. The toxicity was scored before start of the induction regimen, during the CTC chemotherapy, after each course and at the follow-up date. Toxicity was graded according to the National Cancer Institute Common Toxicity Criteria (NCI-CTC) (NCI, 1998). Long-term toxicity was also scored with the NCI-CTC criteria, but because most toxicities were infrequent, they were registered in a dichotomous or descriptive way. Long-term toxicity was defined as toxicity still lasting or appearing more than 1 year after high-dose treatment.

## RESULTS

We have analysed the toxicity of CTC in 100 consecutive patients treated between 1989 and 2000 who received a PBPC-Tx in our institution. At the time of the evaluation, 63 of these 100 patients were still alive. The median follow-up time of all patients was almost 5 years, with a lead follow-up of 137 months.

### Bone marrow recovery

Predictably, the main toxicity of HD-CT was bone marrow suppression. All patients had periods of absolute neutropenia and required platelet and red cell transfusions. As the duration of the neutropenic period and thrombocytopenia is significantly reduced after peripheral stem cell reinfusion with G-CSF compared to the autologous bone marrow transplantation alone, the data of the bone marrow recovery were analysed separately for these patient groups (Table 3

**Table 3:** Bone marrow recovery

	**PBPC-Tx +G-CSF (range)**	**PBPC-Tx (range)**	**ABMT (range)**	**ABMT +G-CSF (range)**
No. of patients	81	10^a^	5	4

Type of tumour				
Breast cancer	76			
Adjuvant		10		
Advanced disease	2			
Germ cell	1		1	1
Other	2		4	3

Day^b^ neutrophil granulocyte count >500^c^	9 (8–20)	18.5 (11–28)	27 (21–29)	18 (14–36)
Day neutrophil granulocyte count >1000^c^	10 (8–20)	20 (17–34)	31 (16–36)	19.5 (16–39)

*Day platelets*				
>50	18 (9–60)	23 (14–39)	27 (2–38)	52 (37–55)
>100	28 (12–120)	25.5 (18–41)	40 (31–56)	53 (44–62)
Time to platelet transfusion independence^d^	13 (8–60)	16 (10–28)	21 (13–27)	28 (27–38)
Day of discharge^e^	13 (10–35)	16.5 (13–19)	20 (17–33)	32 (20–54)
No. of CD34 cells l^−1^ (10^6^ kg^−1^)	9.1 (0.7–35)	10.3 (1.1–12.3)		
No. of CFU-GM (10^4^ kg^−1^)			465 (264–950)	207 (117–404)

*Day fever*>				
38°C	5 (0–18)	3 (0–6)	8 (1–16)	4.5 (0–12)
39°C	2.5 (0–10)	2 (0–4)	3.5 (0–11)	2.5 (0–11)

*No. of transfusions*				
Platelets (occasions)	3 (1–9)	2.5 (2–8)	7 (2–9)	5 (2–9)
Red blood cells (units)	6 (0–35)^f^	6 (4–10)	12 (5–22)	16 (13–22)

*Infections*				
Documented infections	4	1	1	0
Catheter-related infections	9	0	1	2
Suspected infections	58	4	3	0

*Bleeding episode (all)*	20	2	0	4
Epistaxis	7	2		3
Vaginal bleeding	3			
Haematemesis	2			
Haematuria	2			
Gingiva bleeding	2			
Haematomas	2			
Other	2			1
Toxic death	1			

PBPC-Tx=peripheral blood progenitor cell transplantation.

a In two patients G-CSF was started on day 13 after transplantation.

b Day 0 is day of PBPC-Tx.

c Neutrophil granulocyte count × 10^6^ l^−1^.

d Time until platelets are >20 × 10^6^ without platelet transfusion.

e Day 0 is the day of stem cell or bone marrow reinfusion.

f Two patients needed many units of red cells: one patient with a hereditary spherocytosis and one patient with an expanding hematoma.

).

Most patients had PBPC-Tx with G-CSF and the median time to a neutrophil granulocyte count of >500 × 10^6^ l^−1^ was 10 days (range 8–25). The median time to platelet transfusion independence (defined as platelets >20 × 10^9^ l^−1^ without platelet transfusion) was 13 days (range 8–60). The patients required a median of 3 (range 1–9) platelet transfusions and 6 U of red cells (range 2–35). Two patients had an excessive need of red blood cell transfusions: one patient developed a large haematoma in the abdominal wall and ultimately died of multiorgan failure (see below). The second patient required 31 U of red cells because of haemolysis due to a previously undiagnosed hereditary spherocytosis and was later treated successfully with a splenectomy. The patients with combined ABMT and PBPC-Tx had comparable results. As expected, the patients receiving ABMT-only repopulated later (neutrophil count >500 × 10^6^ l^−1^ with G-CSF (*n*=4) on day 18 (range 14–36) and without G-CSF (*n*=5) on day 27 (21–29)), respectively, and the median times to platelet transfusion independence were 21 days (range 13–27) and 28 days (27–38), respectively.

### Infectious complications

In total, 83 patients developed fever ⩾38°C for one or more days (median duration 5 days (range 1–18)) in the neutropenic period following HD-CT (Table 3). Most of these patients (*n*=65) had fever of unknown origin. Five had clinical and radiological signs of pneumonia, but in only one of them a causative microorganism (parainfluenza virus) could be isolated. In this patient, transferal to the intensive care unit was necessary because of respiratory distress. In 12 patients, blood cultures from the indwelling intravenous catheter became positive, usually for coagulase-negative Staphylococci. The amount of positive blood cultures could be relatively high, because in the first courses cultures were performed every day instead of twice a week later on. Six more patients had the following documented infections: one patient had a positive blood culture with a *Streptococcus pneumoniae* without respiratory symptoms. In a second patient, pulmonary tuberculosis was diagnosed on day 8 after PBPC-Tx. A third patient threw up an ascaris worm and received mebendazole for 3 days. A fourth patient had a symptomatic and possibly invasive Candida infection of the mouth. In a fifth patient, a pansinusitis was diagnosed from which *Pseudomonas aeruginosa* was cultured. The sixth patient developed multiorgan failure, possibly resulting from infection of a large haematoma with coagulase-negative Staphylococci (see below).

### Bleeding episodes

Bleeding episodes during low platelet counts were frequent (48%), but were mild and never life-threatening: most frequent were epistaxis (26%) and vaginal bleeding related to the menstrual cycle (12%).

### Non-haematological toxicity

The organ toxicities during the CTC courses and the period thereafter are summarised in Table 4

**Table 4:** Acute toxicity of CTC

**Toxicity**	**Grade (CTC)**	**No. of patients**	**Median day start**	**Median day end**
All patients		100		
Nausea and vomiting	3 or 4	55	−3	6
Diarrhoea	3 or 4	28	0	11
Mucositis	3 or 4	19	2	11
Rash	3 or 4	21	7	13

Ototoxicity				
Hearing loss	1 or 2	10	3	14
	3 or 4	3		
Tinnitus	1 or 2	10	5	15
	3 or 4	3		


Neuropathy	1 or 2	5	28	36
	3 or 4	0		

Haemorrhagic cystitis	1 or 2	1	7	11
	3 or 4			

Cardiotoxicity	1 or 2	3	6	12
	3 or 4	3		

Pulmonary toxicity	1 or 2	5	8	15
	3 or 4	2		

Day 0 is the day of transplantation. CTC was given from day −6 through −3.

. Nausea and vomiting was seen in all patients, despite intensive antiemetic therapy. Many patients (*n*=42) had WHO grade 3–4 nausea and/or vomiting starting on the third day of the CTC course, with a median duration of 8 days. Diarrhaea grade 3–4 was found in 28% of the patients, median starting on day 3 after transplantation and lasting for a median of 5.5 days. Mucositis was usually mild, but grade 3–4 toxicity was found in 19 patients. Skin rash was frequently seen (grade 3–4 toxicity in 21 patients, median starting on day 7 after transplantation) and usually coincided with the administration of broad-spectrum antibiotics.

Symptomatic tinnitus and hearing loss were noted in 12 patients, but only five patients had grade 3–4 toxicity tinnitus and/or hearing loss. At 2 months after transplantation, nine patients (four patients with cisplatin pretreatment) still had symptomatic high-frequency hearing loss and problems with conversation and background noise, eight patients (five patients with cisplatin pretreatment) had intermittent tinnitus and one patient in the breast cancer group still had continuous tinnitus. Audiograms were not routinely performed. Hearing aids were never required. At long-term follow-up, only one patient had symptomatic high-frequency loss and two patients still had symptomatic tinnitus, all in the breast cancer group.

Neuropathy was mild (limited to paresthesias in hands and feet) and occurred in five patients during chemotherapy. After 2 months, nine patients had slight sensory and two patients moderate sensory neuropathy. Neuropathy was more common in the patients with germ cell tumours pretreated with cisplatin than in the breast cancer patients (55 *vs* 7%).

Renal toxicity (defined as a rise of serum creatinine of 1.5 × the normal value (⩾CTC grade 2 toxicity)) was seen in 12 patients and was at least partially reversible in most cases within 2 months (Tables 5

**Table 5:** Liver and renal toxicity of high-dose chemotherapy

	**All patients**	**Day of peak value (median)**
*Alkaline phosphatase*		
⩽5 × normal value	17	4
⩾5 × normal value	1	

*ASAT*		
⩽5 × normal value	80	0
⩾5 × normal value	9	

*ALAT*		
⩽5 × normal value	72	0
⩾5 × normal value	7	

*Bilirubin*		
⩽3 × normal value	35	4
⩾3 × normal value	1	

*Creatinine*		
⩽3 × normal value	32	10
⩾3 × normal value	10	

Day 0 is day of PBPC-Tx.

and 6

**Table 6:** Long-term toxicity

	**Germ cell**	**Breast cancer**	**Other**	**All patients**
No. of patients	8	90	2	**100**

*Ototoxicity*				
*Symptomatic hearing loss*				**1**
High frequency	0	1	0	1
Conversation	0	0	0	0

Intermittent tinnitus	0	2	0	2

*Neuropathy*				**9**
Slight sensory	2^a^	5	0	7
Moderate sensory	0	2	0	2
Slight motor	0	0	0	0

*Cardiotoxicity*				**3**
Myocardial infarction	1	0	0	1
Decline of LV function	0	2	0	2

*Nephrotoxicity*^b^				
Persisting creatinine elevation	3^c^	4	0	7

*Pulmonary toxicity*				
Radiation pneumonitis^d^	0	11	0	**20**
Pleural effusion (transient)	0	6	0	6
Other	1	2	0	3

a LV function=left ventricular function. One patient already had neuropathy before start of the CTC protocol.

b Defined as creatinine >130 *μ*mol l^−1^.

c Three patients already had an elevated creatinine before start of the CTC regimen. One patient needed a kidney transplantation.

d Radiation pneumonitis responsive to steroids. Bold values denote the total number of patients in each category.

). However, seven patients kept a permanent mild elevation of the serum creatinine (CTC grade 1). Three of these patients had a germ cell tumour and had received prior treatment with cisplatin, and all of them already had a creatinine elevation before high-dose therapy. One patient with a germ cell tumour who developed severe kidney toxicity, was found to have a hypoplastic left kidney and nephrosclerosis on the right side, 8 years after HD-CT. Nephrotoxic chemotherapy was thought to be the cause of these problems. He underwent a successful kidney transplantation 8 years after transplantation.

Mild hepatotoxicity (Table 5) was seen in all patients and consisted mainly of a transient rise in ALAT and ASAT on the day of transplant (day 0). A total of 36 patients also had a rise of bilirubin, of alkaline phosphatase (median on day 4) and of gamma GT (median on day 2). In all patients, the liver functions normalised in 1–2 weeks.

Mild haemorrhagic cystitis (WHO grade 2) was seen in one patient between days 7 and 11 after transplant during thrombocytopenia. This did not lead to significant haemorrhagic or haemodynamic complications.

Some degree of cardiac failure during the CTC regimen was observed in six patients and was completely reversible in all cases. All patients developed the first symptoms between days 3 and 8 after transplantation. Symptoms consisted of cardiomegaly (*n*=5), tachycardia (*n*=4), pleural effusion (*n*=2), pericardial effusion (*n*=2) or electrocardiogram changes (*n*=3). All six patients were treated with furosemide and oxygen as needed. One patient was additionally treated with low-dose dopamine during a week and a further patient needed nitroglycerin sublingually.

Seven patients developed pulmonary abnormalities, six with breast cancer and one with a germ cell tumour. The symptoms (dyspnoea, cough and fever) appeared between the second day of the HD-CT until 17 days after transplantation. One patient had a documented parainfluenza pneumonitis and four patients were thought to have a pulmonary infection without identification of a microorganism. As mentioned previously in this paper, one patient reactivated previously unrecognised pulmonary tuberculosis. One patient was diagnosed to have pulmonary embolism and started with anticoagulant therapy. Pleural effusion without cardiac toxicity was seen in one patient possibly due to cyclophosphamide-related toxicity.

All premenopausal female patients became postmenopausal after HD-CT. Psychologic toxicity has been described elsewhere (Schagen *et al*, 2001).

### Toxic death

One toxic death (1%) was seen in the high-risk breast cancer group (Rodenhuis *et al*, 1998). This patient died at day 23 after HD-CT of respiratory distress and multiorgan failure. This patient had been treated with anticoagulant therapy because of thrombosis of the CVC. The coagulation parameters had been normal before the start of anticoagulant therapy. The oral anticoagulation therapy (coumarins) was discontinued before high-dose therapy and low-dose heparin subcutaneously was begun. At 4 days after stopping coumarins, she spontaneously developed an expanding haematoma in the abdominal wall. Laboratory investigations showed a prolonged activated partial prothrombin time (APTT), which could not be explained by the coagulation therapy. Surgery was performed in an effort to localise and stop the bleeding, but this was not successful. She went on to develop severe mucositis and an interstitial pneumonia. No microorganisms were detected at broncho-alveolar lavage. She eventually developed multiorgan failure and the blood/urine/mouth cultures became positive for coagulase-negative Staphylococci despite adequate antibiotic treatment (teicoplanin and later vancomycin). At autopsy, no viable tumour was found but the findings indicated a respiratory distress syndrome and coagulase-negative Staphylococci could still be cultured from the lung.

### Late complications

The long-term toxicities are summarised in Table 6. Nephrotoxicity was seen in seven patients, consisting of a persisting elevation of creatinine (>130 *μ*mol l^−1^). One of these patients, previously treated with cisplatin, developed end-stage renal failure (due to a hypoplastic left kidney and nephrosclerosis of the right kidney) and underwent a successful kidney transplantation, 8 years after PBPC-Tx.

In all, 11 patients with breast cancer who received radiation therapy following HD-CT developed signs of a radiation pneumonitis. All responded readily to corticosteroids. One additional patient had haemoptysis due to a vascular malformation in the lung 4 years after HD-CT and radiotherapy on the mediastinal tumour. He was treated with embolisation and later underwent a lobectomy.

Three patients in the breast cancer group had long-term ototoxicity (symptomatic high-frequency loss (*n*=1), persisting tinnitus (*n*=2)), but no patient including those pretreated with cisplatin had symptomatic hearing deficits. Neuropathy was seen in nine patients, both in the germ cell (*n*=2) and in the breast cancer group (*n*=7). Only one myocardial infarction was seen, not exceeding the number expected for a normal population. Two patients in the breast cancer group, both in the group treated with conventional fluoruracil epirubicin cyclophosphamide (FEC)courses, developed symptomatic cardiac failure 7 and 3 years after HD-CT, respectively. The decline in ejection fraction was thought to be due to the combination of chemotherapy and radiotherapy. At the time of follow-up, both patients were still treated with angiotensin-converting enzyme inhibitors and diuretics with good result.

Irreversible alopecia, which is sometimes seen after multiple cycles of high-dose CTC, never occurred. Herpes zoster infection was seen in 19 patients, all breast cancer patients. This was a rather late complication with a median of 254 days after transplantation (range 28–1060). Four patients had herpes simplex infections, three of them had an anal herpes infection and one patient had an oral herpes infection.

### Second tumours

We found seven second solid neoplasms (Table 7

**Table 7:** Second neoplasms

	All patients
No. of patients	100

*Skin tumours*	**4**
Melanoma	2
Basal cell carcinoma	1
Squamous cell carcinoma	1

Mixed müllerian tumour of ovary	**1**
Cervical polyp of uterus	**1**
Tubular adenoma of colon	**1**

Myelodysplasia	**2**

Bold values denote the total number of patients under each category.

). Two breast cancer patients had benign tumours: a tubular adenoma of the colon and a cervical polyp of the uterus, 5 and 7 years after treatment, respectively. Most tumours were skin tumours. One patient had both a melanoma and a squamous cell carcinoma located at the left upper arm and left thorax wall, respectively, 8 years after the high-dose therapy, both not in irradiated areas. Another melanoma was seen in a patient with germ cell cancer, and a basal cell carcinoma of the mouth was diagnosed in a breast cancer patient 5 and 4 years after treatment, respectively. The only other solid neoplasm was a mixed müllerian tumour of the ovary, 3 years after high-dose therapy.

Two breast cancer patients developed myelodysplasia (MDS). One patient was diagnosed with MDS and had retrospectively already signs of MDS in the bone marrow before the start of chemotherapy. She died of brain metastases of breast cancer, 1 month after developing bone marrow failure due to MDS (8 months after transplant). The second patient developed bone marrow failure almost 8 years after high-dose treatment and died of bone marrow failure due to transformation to an acute leukaemia without having a relapse. The bone marrow of this patient showed multiple cytogenic abnormalities (deletion of 3q, 13q, 20q and addition of 5q and 18q).

## DISCUSSION

We have analysed the clinical data of 100 consecutive patients who underwent HD-CT with CTC. The objective of this analysis was to describe the early and late toxicities of this regimen, which is similar to but not identical with the CTCb (STAMP-V) regimen described by Antman *et al* (1992).

Since the introduction of PBPC-Tx myelosuppression has ceased to be dose-limiting. In patients who also received G-CSF after transplant, the median time to a neutrophil count of ⩾0.5 × 10^9^ l^−1^ was 10 days after transplant and the time to platelet transfusion independence was 13 days after transplant. The brief but profound bone marrow suppression is not without hazards, however, and one of the patients in this series died as a result of bleeding and sepsis. A toxic death rate of 1% is in line with the findings in most recent studies of HD-CT. It is also consistent with the finding of the European Bone Marrow Transplantation registry that a toxic death rate of 1% has been stable in the adjuvant chemotherapy setting for breast cancer since 1995 (Rosti and Ferrante, 2001).

The acute toxicity of CTC is substantial but consistently reversible. We have previously shown that CTC courses can be repeated and we have in fact administered three subsequent CTC courses in patients with breast cancer or germ cell cancer (Rodenhuis *et al*, 1996). Patients who receive more than a single course of CTC may, however, develop major organ toxicity, including severe haemorrhagic cystitis, veno-occlusive disease and haemolytic uraemic syndrome. In addition, in some patients irreversible alopecia may occur. All these problems were never seen in patients who received only a single CTC course, with the exception of the single patient with a very mild haemorrhagic cystitis seen in this series (Table 4).

The supportive care of CTC has been changed throughout the years and we saw the most toxicity in the first courses. Whether this is due to the better supportive care or the increasing experience with HD-CT is unsolved. It would be interesting to compare our results with toxicity data of other centres, but up till now, these data are not available.

An important concern in patients who receive high-dose alkylating therapy is DNA damage of nontumour cells, which may in itself lead to second tumours. In this series of 100 patients, we have observed two cases of myelodysplastic syndrome, one of which was already present before the start of HD-CT. The early MDS may be the result of epirubicin exposure, as seen in the Scandinavian trial (Bergh *et al*, 2000), but in the second patient the interval was too long for a relation with topo-isomerase II inhibitors. There were also four skin cancers (two of which were melanomas) and a mixed müllerian tumour of the ovary (Table 7). Clearly, even a median follow-up of 7 years is insufficient to estimate reliably the number of second solid tumours that may be induced by the treatment.

A second long-term toxicity that we have recently described is impairment of cognitive function (van Dam *et al*, 1998). This type of toxicity has been associated with a single high-dose course CTC and many of the patients reported here have contributed to these studies (Schagen *et al*, 2001). The severity of symptoms associated with these abnormalities at neuropsychological testing is, however, generally mild. Current work focuses on the reversibility of the testing abnormalities after several years.

A variety of high-dose regimens for solid tumours have been reported to date. Some of these have been used in larger randomised studies. The latter regimens can arguably be divided into three categories: (i) the STAMP-I regimen, consisting of cisplatin, BCNU and cyclophosphamide; (ii) the CTCb-like regimens, which are based on cyclophosphamide and thiotepa with or without carboplatin; and (iii) the mitoxantrone-based regimens. We believe that the first category, the STAMP-I regimen, is too toxic for routine use. The two studies (Peters *et al*, 1993; Nikcevich *et al*, 2002) that employed this carmustine-based combination reported high toxic death rates due to organ toxicity, mainly interstitial pneumonitis. Even very experienced centres that optimally exploit corticosteroids at the earliest sign of pulmonary symptoms report a 5% toxic death rate (Antman *et al*, 1992).

Regimens of the third category, employing mitoxantrone, may have acceptable toxicity and appear to be effective in breast cancer, at least in preliminary analyses of randomised studies (Roche *et al*, 2001). It will be important to evaluate the long-term cardiotoxicity of high-dose mitoxantrone, particularly in patients who have previously received anthracyclines for breast cancer and who subsequently undergo radiation therapy of the chest wall, which includes the myocardium.

The CTCb-like regimens have been reported to be well-tolerated. We have extensively studied the complicated pharmacokinetics of this combination and have shown that the activation route of the prodrug cyclophosphamide is effectively inhibited in the presence of thiotepa. In theory, a continuous infusion of thiotepa could lead to less active second metabolites, to less toxicity and possibly even less efficacy of the cyclophosphamide–thiotepa combination (Anderson *et al*, 1996; Peters *et al*, 1999; Teicher *et al*, 1999; Huitema *et al*, 2000). In the classic STAMP-V or CTCb regimen, cyclophosphamide and thiotepa are also being given as continuous infusions, but this is different in the CTC regimen reported in this paper. Perhaps this difference could account for the possibly somewhat higher acute toxicity of CTC in comparison to CTCb. This difference could, of course, also arise from the substantially higher carboplatin dose in the CTC regimen. We have not been able to administer CTC on an outpatient basis, mainly because of nausea and vomiting. It is interesting that this type of toxicity is much less pronounced when the dose of CTC is decreased by one-third (Schrama *et al*, 2001). With such a dose reduction, management of the aplastic phase outside the hospital becomes feasible (Westermann *et al*, 1999). This illustrates that a relatively minor difference in drug exposure can significantly affect the tolerance of the regimen.

At this point in time, the future of HD-CT in the curative treatment of solid tumours remains unclear. Over 3000 patients with high-risk breast cancer have now been treated in randomised studies and most of these studies show a modest benefit for the high-dose arm in preliminary analyses. It is therefore likely that certain individual tumours that cannot be cured by standard-dose chemotherapy may sometimes be eradicated by high-dose therapy. Since the latter treatment modality is toxic and expensive, the ability to select tumours specifically for this treatment modality would clearly be extremely useful. The recent development of messenger RNA expression profiling (Van‘t Veer *et al*, 2002) has raised hope that this technique could be used to test breast cancers and other malignancies for important biological characteristics such as alkylating agent sensitivity.

At the same time, efforts are continuing to decrease the toxicity of HD-CT. We have recently developed assays that allow us to analyse the pharmacokinetics and pharmacodynamics of the three high-dose agents in the CTC regimen, including the major metabolites of cyclophosphamide. As expected, major differences exist between patients in the AUCs of cyclophosphamide metabolites, thiotepa and its major metabolite tepa. We and others (Wolff *et al*, 1990; Huitema *et al*, 2001) have observed that a significant relation exists between the elevation of liver enzymes and the combined thiotepa and tepa AUCs. In addition, it is mainly the AUC of thiotepa and tepa that is associated with mucositis (Hussein *et al*, 1996; Przepiorka *et al*, 1995. We are currently employing the strategy of therapeutic drug monitoring, in which pharmacokinetic measurements on the first day of administration of CTC are used to determine the dosage of the three agents on days 3 and 4. In this way, it is possible to prevent excessive exposure to drugs or their metabolites in some patients and to increase exposure in patients with unexpectedly rapid drug disposition (Huitema *et al*, 2001, 2002). It remains to be shown, however, that such a fine tuning of dose leads to less toxicity, improved efficacy, or both.

The precise role for high-dose alkylating chemotherapy in solid tumours is now gradually being defined. We believe that the CTC regimen described in this paper is a suitable combination to be further tested in randomised clinical trials.
